# Colistin for lung infection: an update

**DOI:** 10.1186/s40560-015-0072-9

**Published:** 2015-01-22

**Authors:** Mohan Gurjar

**Affiliations:** Department of Critical Care Medicine, Sanjay Gandhi Postgraduate Institute of Medical Sciences (SGPGIMS), Lucknow, UP India

**Keywords:** Colistin, Lung infection, Pneumonia, Ventilator-associated pneumonia, Critically ill

## Abstract

Increasing incidence of resistance of gram-negative bacteria against even newer antibiotic including carbapenem has generated interest in the old antibiotic colistin, which are being used as salvage therapy in the treatment of multidrug resistant infection. Colistin has excellent bactericidal activity against most gram-negative bacilli. It has shown persist level in the liver, kidney, heart, and muscle; while it is poorly distributed to the bones, cerebrospinal fluid, lung parenchyma, and pleural cavity. Being an old drug, colistin was never gone through the drug development process needed for compliance with competent regulatory authorities that resulted in very much limited understanding of pharmacokinetic (PK) and pharmacodynamic (PD) parameters, such as *C*_max_/MIC ratio, AUC/MIC and T > MIC that could predict the efficacy of colistin. In available PK/PD studies of colistin, mean maximum serum concentration (*C*_max_) of colistin were found just above the MIC breakpoint at steady states that would most probably lead to suboptimal for killing the bacteria, even at dosages of 3.0 million international units (MIU) i.e., 240 mg of colistimethate sodium (CMS) intravenously every 8 h. These finding stresses to use high loading as well as high maintenance dose of intravenous colistin. It is not only suboptimal plasma concentration of colistin but also poor lung tissue concentration, which has been demonstrated in recent studies, poses major concern in using intravenous colistin. Combination therapy mainly with carbapenems shows synergistic effect. In recent studies, inhaled colistin has been found promising in treatment of lung infection due to MDR gram-negative bacteria. New evidence shows less toxicity than previously reported.

## Introduction

Worldwide, there are growing threats to modern medicine from the emergence of multidrug resistant (MDR) bacteria causing nosocomial infection. This coupled with marked decline in the discovery and development of novel antibiotics especially against gram-negative bacteria in the last two decades leads to critical challenge to clinicians. MDR bacteria are usually defined as when it is resistant to three or more group of antibiotics. In clinical practice, antibiotics commonly used in treating gram-negative infection are penicillin, cephalosporin, carbapenem, monobactem, quinolone, and aminoglycoside. Increasing incidence of resistance of gram-negative bacteria against even newer antibiotic including carbapenem has been reported in many countries. In the last few years, with a paucity of available antibiotic option, interest has been renewed in the old antibiotic, polymyxins, as salvage therapy in the management of infections caused by MDR gram-negative pathogens including *Pseudomonas, Acinetobacter, Klebsiella,* and *Enterobacter* species [1-3].

Polymyxins are group of cationic polypeptide antibiotics, having five different compounds (polymyxin A-E) [4]. In 1949, colistin (Polymyxin E) was first time isolated from *Bacillus polymyxa* var. *colistinus* by Koyama Y. and colleagues in Japan (Table 1) [5]. Only polymyxin E (Colistin) and polymyxin B have been used in clinical practice since their discovery [1,2]. Both, colistin and polymyxib B, are produced by the soil bacterium *Bacillus* spp., and they differ in structure only by one amino acid at position 6 (-Leu in colistin, while -Phe in polymyxin B) [4].

**Table 1:** **History of colistin**

**Year**	**Event**
1947	Discovery of polymyxins from bacteria *Paenibacillus polymyxa*
1949	Colistin (polymyxin E) was first time isolated from *Bacillus polymyxa* var. *colistinus* by Koyama Y. and colleagues in Japan
1959	Colistin became available in intravenous formulation (as colistimethate sodium) for clinical uses
1960s–1970s	Colistin used for gram-negative infection; later on, uses decline due to its toxicities
1990s–2000s	Used mainly for lung infection due to MDR gram-negative pathogens in patients with cystic fibrosis
2000 onwards	Currently in use to treat healthcare-associated MDR gram-negative infection

Clinical experience is more with colistin in comparison to polymyxin B due to its wider use. After introduction in clinical practice, uses of parenteral colistin were gradually waned worldwide within two decades due to reported severe toxicities, while its topical uses continued [2-4]. So, era of colistin as intravenous use could be divided in three phases: 1950–1970: against gram-negative infection; 1990–2000: for MDR gram-negative pathogens in cystic fibrosis; and 2000 onwards: nosocomial infection due to MDR gram-negative pathogens. In this presented descriptive review, we looked in published literature for the use of colistin in lung infection in critically ill patients without cystic fibrosis.

## Review

### Current issues in colistin therapy

**Figure 1:**
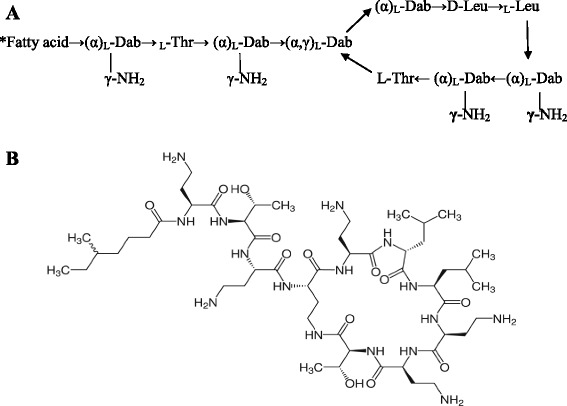
**Descriptive and chemical structure of colistin. (A)** Descriptive **s**tructure of colistin [*Fatty acid: (S)-6-methyloctanoic acid for colistin A, (S)-6-methylheptanoic acid for colistin B; *Thr* threonine, *Leu* leucine, *Dab* α,γ-diaminobutyric acid]. **(B)** Chemical structure of colistin.

Colistin, itself is composed of mixture of closely related components, mainly colistin A (polymyxin E1) and colistin B (polymyxin E2), which are acylated by (S)-6-methyloctanoic acid and (S)-6-methylheptanoic acid, respectively (Figure 1) [4]. Each molecule has a cationic polypeptide ring with a lipophilic fatty acid chain.

### Spectrum of activity

Colistin has excellent bactericidal activity against most gram-negative bacilli, while no activity against all gram-positive and most anaerobes (except few like *Prevotella* spp., *Fusobacterium* spp.) organisms (Table 2) [6]. Colistin has also been reported to be potentially active against several *Mycobacterium* spp. [7].

**Table 2:** **Spectrum of activity of colistin**

**Gram-negative bacteria**		
**Susceptible**	**Resistant**	**Variable**
Gram-negative bacilli: *Pseudomonas aeruginosa*, *Acinetobacter* spp., *Escherichia coli*, *Klebsiella* spp., *Enterobacter* spp., *Citrobacter* spp., *Salmonella* spp., *Shigella* spp., *Haemophilus influenza*, *Bordetella pertusis*, *Legionella pneumophila*	Gram-negative bacilli: *Proteus* spp., *Providencia* spp., *Morganella morgani*, *Serratia* spp., *Edwardsiella tarda*, *Burkholderia* spp., *Brucella* spp.	Gram-negative bacilli: *Stenotrophomonas maltophilia*, *Aeromonas* spp., *Vibrio* spp.
	Gram-negative cocci: *Neisseria gonorrheae*, *Neisseria meningitides*, *Moraxella catarrhalis*	

### Mechanism of action and resistance

The targets site for colistin is lipopolysaccharide (LPS) component of the outer membrane of gram-negative bacteria. In there, colistin interacts with the lipid A component of the LPS, displacing the calcium and magnesium bridges that stabilize the LPS, leading to permeabilizing the bacterial outer membrane that can be described as detergent-like mechanism of action [4]. Subsequently, colistin inserted through these cracks in the outer membrane of bacteria causes ‘self-promoted uptake’ as well as disruption of the integrity of the inner membrane also that leads to bacterial killing [1,4].

By another postulated mechanism, polycationic polymyxin binds to anionic phospholipid; this contact promotes lipid exchange between inner and outer membrane results in the loss of phospholipid composition leading to an osmotic imbalance resulting in lytic cell death [4].

The majority of mechanisms of resistance to colistin are based on modifications to the lipid A portion of LPS of gram-negative bacteria, like *Pseudomonas aeruginosa* and *Escherichia coli*, that reduces its net negative charge resulting in less electrostatic interaction with positively charged colistin molecule [1,4]. While in *Klebsiella pneumoniae*, the mechanism of resistance to colistin is because of increased production of capsule polysaccharide [8].

### Susceptibility test and heteroresistance

Antimicrobial susceptibility testing for colistin can be performed using either diffusion (disc diffusion, prediffusion test, or epsilometer test (E-test) or dilution techniques (broth microdilution or agar dilution) [6]. Colistin sulfate is recommended in susceptibility testing instead of colistimethate sodium (CMS) [2,6]. The reason for not using CMS in susceptibility testing, which is used clinically (see below), is an inactive pro-drug and also undergo variable hydrolysis resulting in differential killing characteristics and varying susceptibility report. There is another issue that colistin has, that because of its large molecule, it diffuses inadequately into the medium which makes disc diffusion test as a poor performer in susceptibility test. Among all these methods, broth microdilution and agar dilution methods are considered as reference methods [6,9]. Alternatively, E-test method is a reliable, easy to perform, and less time-consuming [6].

The breakpoints for colistin susceptibility are defined differently by two main societies: as per US Clinical and Laboratory Standards Institution (CLSI), ≤2 mg/L as the susceptibility breakpoint and >2 mg/L as the resistance breakpoint, while as per British Society for Antimicrobial Chemotherapy (BSAC), ≤4 mg/L as susceptible and ≥8 mg/L as resistant [2].

In a population analysis, profiles of ‘colistin-susceptible’ clinical isolates *Acinetobacter baumannii* showed that subpopulation <0.1% bacteria grew in the presence of colistin 3 to 10 μg/ml, where minimum inhibitory concentration (MIC) of colistin against all isolates were within 0.25 to 2 μg/ml [10]. This represents the heterogeneous colistin-resistant in colistin-susceptible clinical isolates called ‘heteroresistance’. This heteroresistance is observed more frequently in MDR *A. baumannii* and less in MDR *P. aeruginosa.* This heteroresistance phenomenon explains the substantial regrowth of *A. baumannii* occurring at 24 h even at colistin concentrations up to 64 × MIC [10]. This heteroresistance also suggests that colistin monotherapy and extended interval dosage regimens may be problematic especially during treatment of *A. baumannii*.

### Commercially available forms

Commercially, there are two available forms of colistin (Table 3): colistin sulfate for oral and topical use and CMS for parenteral use, as it is less toxic than colistin sulfate [1-3].

**Table 3:** **Comparison of two different salts of colistin**

	**Colistimethate sodium**	**Colistin sulfate**
Prepared from	Colistin (chemically modified)	Synthesized non-ribosomally from *Bacillus polymyxa* subspecie*s colistinus*
Salt	Sodium	Sulfate
Chemically	Polyanion	Polycation
Stability	Less	More
Elimination	Renal	Non-renal
Half-life	~2 h	~4 h
Anti-microbial activity	Non-active prodrug: 32 different products	Active
Available for	Parenteral use, inhalation use	Oral and topical use, inhalation use

Colistimethate sodium, available as dry powder for reconstitution before administration, is marketed differently with respect to the content in the vial as well as recommended dose. Few brands (Colomycin, Coly-Monas, Xylistin) labeled the content as international unit, IU, (0.5, 1, or 2 million international units (MIU) per vial), while other (Coly-Mycin M) labeled the content as mg of colistin base activity (150 mg per vial) [2]. So, the clinician and researcher should be well aware about this product discrepancy as regards to dose content per vial by different brands, and prescription/description should be very much clear about dose as mg or IU of either CMS or colistin base activity.

Colistin activity equivalent

1 mg of CMS = 0.375 mg of colistin base activity = 12,500 IU

1 mg of colistin base activity = 2.6 mg of CMS = 32,500 IU

### Pharmacokinetic/pharmacodynamic and optimal dosing of intravenous colistin

CMS as not being stable both *in vivo* and *vitro* is hydrolysed to many derivatives including colistin and also makes it difficult to measure accurately both CMS and colistin in biological samples [2]. Available literature till now are not much clear about pharmacokinetics of colistin, especially in patient with various degrees of renal impairment [11]. Absorption from oral mucosa or gastrointestinal tract does not occur. After parenteral administration, colistin achieves low protein binding, approximately 50%. About two thirds of CMS is eliminated as unchanged mainly by the renal route within 24 h [1,2]. On the other hand, elimination of colistin is non-renal and non-biliary route by unknown mechanism [2,11]. It has shown persist level in the liver, kidney, heart, and muscle, while it is poorly distributed to the bones, cerebrospinal fluid, lung parenchyma, and pleural cavity [1-3,11].

In most of the published literature, intravenous colistin were used in doses of 2.5 to 5.0 mg/kg/day in two to four divided doses in patients with normal renal function and also without clear mentioning of drug used in mg of either CMS or colistin base activity. Being an old drug, colistin was never gone through the drug development process needed for compliance with competent regulatory authorities that resulted in very much limited understanding of PK and PD parameters, such as *C*_max_/MIC ratio, AUC/MIC, and T > MIC that could predict the efficacy of colistin [2,11]. Recent studies started to reveal about PK/PD of colistin and also demonstrate that it has poor postantibiotic effect (PAE) [11].

In PK/PD studies of colistin, mean maximum serum concentration (*C*_max_) of colistin were found just above the MIC breakpoint of 2 mg/L (Clinical and Laboratory Standards Institute) at steady states that would most probably lead to suboptimal for killing the bacteria, even at dosages of 3.0 MIU i.e., 240 mg of CMS intravenously every 8 h [12-14]. Steady state concentration was achieved at least after 48 h of starting the intravenous colistin [12]. In a population of critically ill patients where pharmacokinetic analysis of colistin was done after intravenous administration, Plachouras et al. predicted that plasma colistin concentration on the basis of the model developed for different dosage regimen revealed that loading dose of 9 or 12 MIU of CMS as infusion of 15 min or 2 h with maintenance dose of 4.5 MIU CMS every 12 h achieves rapid (<12 h) and higher plasma colistin in comparison to dosing regimen 3 MIU as a 15-min infusion of CMS every 8 h, where it takes about 48 h to reach 2 mg/L [14]. Daikos et al. also studied the three different daily doses of CMS (3 MIU every 8 h, 4.5 MIU every 12 h, and 9 MIU every 24 h) that achieved mean serum concentration (*C*_max_) of colistin 3.34, 2.98, and 5.63 μg/ml, respectively, and found that all serum samples containing colistin >4 μg/ml killed *P. aeruginosa*, whereas only 40% of samples containing colistin <4 μg/ml results in complete eradication of *P. aeruginosa* (having MIC 1 μg/ml) [13]. But at present, studies evaluating clinical outcome with these high dosage of colistin are lacking.

In a recent study of PK/PD of colistin, 2 MIU every 8 h, in critically ill patients, Karnik ND et al. observed that the mean (range) of the maximum plasma drug concentration/minimum inhibitory concentration (*C*_max_/MIC) ratio for *Acinetobacter* spp. was 26.3 (0.9–64.9) at steady state, while for *Pseudomonas* spp., it was 3.82 (2.3–10.9) [15]. This shows that an optimum value of the *C*_max_/MIC ratio of >8 was achieved against *Acinetobacter*, not for *Pseudomonas*. Bergen et al. examined the PK/PD relationship of colistin against *P. aeruginosa* and found that colistin efficacy against *P. aeruginosa* was best correlated with the AUC/MIC ratio of total and unbound colistin rather than the *C*_max_/MIC ratio [16].

It is not only the optimal plasma concentration but the site of infection also defines the optimal antimicrobial therapy. Interestingly, in a study, Imberti et al. found undetectable level of colistin in broncho-alveolar lavage fluid of critically ill patients at steady state mean plasma colistin maximum concentration of 2.21 μg/ml after the intravenous administration of CMS 2 MIU every 8 h for at least 2 days [17].

### Use in patients with renal dysfunction

Despite colistin is eliminated by non-renal route, dose adjustment for renal dysfunction is necessary, as there is reduced renal dependent elimination of its pro-drug CMS which ultimately lead to increased colistin level [2,11]. Despite scarcity of studies of PK/PD of colistin in patients with renal failure, recent recommended doses are [2]:

- Serum creatinine level 1.3–1.5 mg/dl: 2 MIU (160 mg) of CMS every 8 h
- Serum creatinine level 1.6–2.5 mg/dl: 2 MIU (160 mg) of CMS every 12 h
- Serum creatinine level ≥2.6 mg/dl: 2 MIU (160 mg) of CMS every 24 h

Patient on renal replacement therapy:

- 2 MIU (160 mg) of CMS after each hemodialysis
- 2 MIU (160 mg) of CMS daily during peritoneal dialysis

In a study about pharmacokinetics of colistin in critically ill patients receiving continuous venovenous hemodiafiltration (CRRT), Karvanen M et al. suggested that dose regimen of 2 MIU CMS every 8 h is inadequate [18]. In another recently published study, Honore et al. postulate from their experience that patients undergoing CRRT may receive substantially higher doses of colistin, i.e., high loading dose followed by a maintenance dose of up to 4.5 MIU every 8 h because the drug is continuously filtered and also significantly adsorbed in the bulk of the dialysis membrane [19].

### Inhaled colistin

For aerosol inhalation purpose, both forms of colistin (colistin sulfate and CMS) could be used [2,3]. Colistin, in the same intravenous formulation, is dissolved in 4–6 ml of normal saline or sterile water to deliver through nebulizer, though this is not approved by the Food and Drug Administration (FDA) [20,21]. There are few studies on the PK of colistin after inhalation. Ratjen et al. evaluated the colistin pharmacokinetics postinhalation in patients with cystic fibrosis [22]. This study finds that a single dose of CMS (2 MIU) achieve significant higher drug concentration in the sputum even after 12 h with low level in serum and urine. In another experimental study done by Lu et al. where pneumonia caused by *P. aeruginosa* in piglets and CMS was administered either by nebulization every 12 h or intravenous every 8 h, lung tissue concentration of colistin was measured [23]. Colistin was found undetected in the lung tissue after intravenous infusion, while after nebulization, peak lung tissue concentrations were significantly higher in the lung segments (more in mild pneumonia segments and less in severe pneumonia area, median 10.0 versus 1.2 μg/g).

As per drug package insert information, the recommended doses of colistin when given by inhalation are as below [24]:

- Body weight <40 kg: 0.5 MIU (40 mg) of CMS every 12 h
- Body weight >40 kg: 1.0 MIU (80 mg) of CMS every 12 h
- For recurrent or severe pulmonary infection: 2.0 MIU (160 mg) of CMS every 8 h

Optimal inhalation therapy also requires consideration of several factors like the type of nebulizer, aerosol particle size, position of patient, severity of airway obstruction, etc. In a mechanically ventilated patient, there are factors more than this, i.e., artificial airway size, humidity, gas density, tidal volume, nebulization cycling during inspiration versus continuous, etc., which may affect drug delivery at the target site [25].

### Combined therapy with other antibiotics and synergism

In clinical practice, colistin is frequently used as combination therapy, though there is scarcity of data that whether combination therapy is superior to monotherapy. One of the reasons for limited studies may be using this drug in treating MDR pathogens that deterred the clinician from providing colistin as a monotherapy. In a recent review by Petrosillo et al. on colistin monotherapy versus combination therapy in animal and clinical studies, they revealed that synergistic effect was detected in all the nine studies examining the combination of colistin and rifampicin, whereas carbapenems exhibited a synergistic effect in two out of three studies [26]. Colistin combined with tigecycline did not show good synergistic action [27]. Considerable synergy was found between levofloxacin and colistin in a recently published study [28]. Pankuch et al. studied time killing synergy with subinhibitory concentration of meropenem and colistin and found significant synergy against *A. baumannii* at 6, 12, and 24 h, while there was no significant synergy against *P. aeruginosa* at any point of time [29]. In another *in vitro* study by Souli et al., the combination of imipenem (irrespective of MIC) and colistin against *K. pneumoniae* showed synergistic activity against isolates susceptible either to both agents or to colistin, while rarely synergistic against non-colistin-susceptible strains [30]. A recently published study by Leu et al. showed that the synergy of imipenem and colistin against imipenem-non-susceptible multi-drug resistant *A. baumannii* was significantly better for the colistin concentration at 1 mg/L than that at 0.5 mg/L [31].

### Toxicity

Two types of toxicity, namely, nephrotoxicity and neurotoxicity has been reported with the uses of colistin. A systematic review of the toxicity of polymyxin revealed that in the old literature, incidences of both toxicities were reported to be considerably high, while new evidence shows less toxicity than previously reported [32]. The observed nephrotoxicity was as high as 50% in old studies versus 15%–25% in recent studies, although the definition of nephrotoxicity was not standardized between the studies [32]. Renal toxicity of colistin has been described as dose dependent and may be partly due to their D-amino acid content and fatty acid component that leads to acute tubular necrosis. Renal toxicity from colistin has been found apparently less than the use of polymyxin B [32]. Age (more than 60 years) has also been found as a significant risk for colistin-induced nephrotoxicity [33]. The concurrent administration of nephrotoxic drugs, hypovolemia or shock, and severity of illness may increase the likelihood of development of acute kidney injury (AKI).

In a recent preliminary study on high-dose colistin (9 MIU twice-daily), administration in critically ill patients found that AKI developed during 18% of treatment courses did not require renal replacement therapy (RRT) and subsided within 10 days from colistin discontinuation [34]. Also, there was no correlation between variations in serum creatinine level and daily and cumulative doses of colistin or duration of colistin treatment. In another study, Dewan et al. also reported AKI in 16% of patients in whom high dose, extended interval colistin (9 MIU stat followed by 4.5 MIU 12 hourly) was used, while no patient required RRT [35].

Overall incidence of neurotoxicity related to colistin use is less than the nephrotoxicity. Earlier studies reported paresthesias in about one forth of patients receiving colistin, with few case reports of neuromuscular blockade or apnea, while recent studies did not reported any significant neurotoxicity [32,36]. Neurotoxicity is also dose dependent and may be triggered by the presence of risk factors like the presence of hypoxia, co-administration of muscle relaxant, narcotics, sedatives, or steroids [32,37,38].

Colistin aerosol inhalation therapy is generally well tolerated with few reported side effects like throat irritation, cough, and bronchospasm, which may be because of the presence of excipients/preservatives [20].

### Colistin for lung infection and clinical outcome

In the last decade, many studies published the use of colistin in the treatment of lung infection in patients without cystic fibrosis, with conflicting results. Most of the studies have population of adult ICU patients with MDR VAP, and types of study were single-arm (Table 4) [11,39-75]. In a systematic review and meta-regression to know the efficacy of colistin for the treatment of VAP, Florescu et al. analyzed 6 controlled studies (359 patients) and single-arm analysis from 14 studies (437 patients) [76]. Among the six two-arm studies (three prospective including one RCT and three retrospective studies), which were unblinded, the mean duration of intravenous colistin was 11.4 days at a mean dose of 252.5 mg/70 kg/day and aerosolized colistin was administered for a mean 9.25 days at a mean dose of 355 mg/70 kg/day. While, in single-arm studies, intravenous colistin was used for a mean of 15 days at a mean dose of 209.5 mg/70 kg/day and aerosolized form was given for a mean of 14 days at a mean dose of 80 mg/70 kg/day. In the meta-analysis of six controlled studies, there was no significant difference for overall clinical response between colistin and control groups (OR 1.14 [95% CI 0.74–1.77; *p* = 0.56]; *I*2 = 0%; *Q* = 4.98 [*p* = 0.42]), even after controlling for concomitant antibiotic treatment, or for the dose of intravenous colistin. Though, there was a trend for better microbiological response. Also, there was no significant difference between the colistin and control groups with respect to ICU, hospital, or 28-day mortality. Neither, incidence of nephrotoxicity was different in both groups; but one study reported higher incidence for respiratory toxicity [55]. In the meta-analysis of single-arm studies, there was favorable clinical response with colistin found (95% CI 0.64–0.80; *Q* = 55.3; *p* < 0.0001). Also, there was a significant microbiological response for aerosolized form, but not with intravenous form. There was a higher incidence of nephrotoxicity with intravenous administration, and no patient had neurotoxicity or respiratory side effects. These single-arm studies also showed mortality benefit (ICU, hospital, as well as 28-day mortality) in this meta-analysis [76].

**Table 4:** **Recent studies on colistin for the treatment of ventilator-associated pneumonia (VAP)**

**Year**	**Prospective or retrospective**	**Randomized controlled trial**	**Studied patients with VAP**	**Organism isolated**	**Route of administration**	**Country**	**First author [reference]**
2003	Prospective	No	35	*Acinetobacter baumanii*	IV	Spain	Garnacho-Montero [39]
2003	Prospective	No	18	*Pseudomonas aeruginosa*	IV	USA	Linden [40]
2003	Prospective	No	15	*P. aeruginosa*, *A. baumanii*	IV	Greece	Markou [41]
2006	Prospective	No	9	*P. aeruginosa*, *A. baumanii*	IV and AS	Greece	Falagas [42]
2006	Prospective	No	16	*A. baumanii*	AS	Morocco	Motaouakkil [43]
2007	Retrospective	No	120	*P. aeruginosa*, *A. baumanii*	IV	Tunisia	Kallel [44]
2007	Retrospective	No	61	*P. aeruginosa*, *A. baumanii*	IV	Argentina	Rios [45]
2008	Prospective	Yes	28	*A. baumanii*	IV	Greece	Betrosian [46]
2008	Retrospective	No	10	*A. baumanii*	IV	Korea	Song [47]
2008	Prospective	No	8	*P. aeruginosa*	IV and AS	Greece	Mastoraki [48]
2008	Prospective	No	19	*A. baumanii*	IV	Italy	Bassetti [49]
2008	Prospective	No	60	*P. aeruginosa*, *A. baumanii*, *Klebseilla pneumoniae*	AS	Greece	Michalopoulos [50]
2008	Prospective	No	10	*P. aeruginosa*, *A. baumanii*	IV	Greece	Markou [11]
2009	Retrospective	No	9	*P. aeruginosa*, *A. baumanii*	IV	Turkey	Tasbakan [51]
2009	Retrospective	No	41	*A. baumanii*	IV	South Korea	Jang [52]
2010	Retrospective	No	86	*P. aeruginosa*, *A. baumanii*, *K. pneumoniae*	IV and AS	Greece	Kofteridis [53]
2010	Retrospective	No	121	*P. aeruginosa*, *A. baumanii*, *K. pneumoniae*	IV and AS	Greece	Korbila [54]
2010	Prospective	Yes	100	*P. aeruginosa, A. baumanii*, *K. pneumonia*, *Escherichia coli*	AS	Thailand	Rattanaumpawan [55]
2010	Retrospective	No	45	*A. baumanii*	AS	Taiwan	Lin [56]
2010	Retrospective	No	11	*P. aeruginosa*, *A. baumanii*, *K. pneumonia*, *Enterobacter cloacae*	IV	Greece	Iosifidis [57]
2011	Retrospective	No	15	*A. baumanii*	AS	Thailand	Nakwan [58]
2011	Prospective	No	15	*A. baumanii*	IV and AS	Spain	Perez-Pedrero [59]
2011	Retrospective	No	20	*P. aeruginosa*	IV and AS	Belgium	Naesena [60]
2012	Prospective	No	165	*P. aeruginosa*, *A. baumanii*	AS	France	Lu [61]
2012	Retrospective	No	36	*A. baumanii*	IV	Turkey	Simsek [62]
2012	Retrospective	No	45	*A. baumanii*	IV and AS	Turkey	Kalin [63]
2013	Retrospective	No	98	*A. baumanii*	IV	Israel	Zalts [64]
2013	Retrospective	No	208	*P. aeruginosa, A. baumanii*, *K. pneumonia*	IV and AS	Italy	Tumbarello [65]
2013	Prospective	Yes	43	*A. baumanii*	IV	Turkey	Aydemir [66]
2013	Retrospective	No	95	*P. aeruginosa*, *A. baumanii*, *K. pneumonia*	IV and AS	USA	Doshi [67]
2013	Retrospective	No	49	*A. baumanii*	IV and AS	Spain	Garnacho-Montero [68]
2014	Retrospective	No	82	*A. baumanii*	IV	Turkey	Kalin [69]
2014	Retrospective	No	26	*A. baumanii*	AS	Taiwan	Hsieh [70]
2014	Retrospective	No	130	*A. baumanii*	IV	Thailand	Khawcharoenporn [71]
2014	Retrospective	No	118	*A. baumanii*	IV	Taiwan	Chuang [72]
2014	Retrospective	No	141	*A. baumanii*, *P. aeruginosa Enterobacteriaceae*	IV	France	Soubirou [73]
2014	Retrospective	No	10	*A. baumanii*	AS	Korea	Choi [74]
2014	Retrospective	No	107	*P. aeruginosa*, *A. baumanii*	IV	Italy	Petrosillo [75]

*IV* intravenous, *AS* aerosolized.

In another recently published systematic review and meta-analysis of colistin for the treatment of VAP caused by MDR gram-negative bacteria, Gu et al. included 14 studies (published during 2003 to 2013, total 1,167 patients), 2 were RCTs, 4 were case-control studies, and 8 were cohort studies [77]. In this analysis for use of colistin versus β-lactam antibiotics, they found that there was no significant difference in both groups for clinical cure rate (OR = 1, 95% CI 0.68–1.47, *p* = 0.99, *I*2 = 0%), microbiological eradication, ICU mortality, hospital mortality, and nephrotoxicity. While comparing aerosolized plus intravenous colistin (AS plus IV colistin) versus intravenous colistin alone, this meta-analysis revealed that AS plus IV colistin had a higher clinical cure rate (OR = 2.12, 95% CI 1.40–3.20, *p* = 0.0004, *I*2 = 0%), but there was no significant differences in microbiological eradication, ICU, and hospital mortality and nephrotoxicity between both (AS plus IV colistin and intravenous colistin alone) groups. This meta-analysis also found that colistin-combined therapy did not have a better clinical cure, microbilogical eradication, ICU and hospital mortality, or nephrotoxicity, when compared with colistin monotherapy, for the treatment of MDR gram-negative VAP [77].

While another systematic review and meta-analysis on the role of aerosolized colistin in the treatment of VAP, Valachis et al. included 8 studies (690 patients) and found a statistically significant improvement in clinical response as well as microbiological eradication, when AS colistin was added to the standard antimicrobial therapy in comparison with patients who received IV colistin (OR, 1.57; 95% CI, 1.14–2.15; *p* = 0.006 and OR, 1.61; 95% CI, 1.11–2.35; *p* = 0.01, respectively) [78]. Overall mortality was not found to be significantly different between the two comparative arms (OR, 0.74; 95% CI, 0.54–1.01; p = 0.06). When AS colistin monotherapy was compared with IV colistin (31 patients, 2 studies), there was no differences between the two treatment arms for clinical and microbiological eradication. The authors have also commented that the quality of evidences for the outcomes was very low according to the Grading of Recommendations Assessment, Development, and Evaluation (GRADE) approach [78].

All three meta-analyses of colistin for the treatment of VAP are summarized in Table 5.

**Table 5:** **Summary of 3 meta-analysis of colistin for the treatment of ventilator-associated pneumonia**

**Meta-analysis**	**Outcome**		
	**Clinical cure**	**Microbiological eradication**	**ICU mortality**
A. Florescu et al. [76]			
Colistin versus control group	Not significant; 6 two-arm studies, 359 patients (OR 1.14 [95% CI 0.74–1.77; *p* = 0.56]; *I*2 = 0%; *Q* = 4.98 [*p* = 0.42])	Not significant; 2 studies, 128 patients (OR 1.997 [95% CI 0.97–4.12; *p* = 0.06]; *I*2 = 2.48%; *Q* = 1.03 [*p* = 0.31])	Not significant; 2 studies, 155 patients (OR 1.27 [95% CI 0.66–2.43; *p* = 0.47]; *I*2 = 0%; *Q* = 0.057 [*p* = 0.81])
Single-arm studies	Significant; 13 studies, 429 patients (95% CI 0.64–0.80; *Q* = 55.3; *p* < 0.0001)	Significant; 9 studies, 267 patients (95% CI 0.44–0.79; *Q* = 121.56; *p* < 0.0001)	Significant; 5 studies, 257 patients (95% CI 0.15–0.42; *Q* = 43.61; *p* < 0.0001)
B. Gu et al. [77]			
Colistin versus β-Lactam antibiotics	Not significant; 6 studies, 507 patients (OR = 1, 95% CI 0.68–1.47, *p* = 0.99, *I*2 = 0%)	Not significant; 3 studies, 91 patients (OR = 0.64, 95% CI 0.18–2.22, *p* = 0.48, *I*2 = 38%)	Not significant; 3 studies, 320 patients; (OR = 1.02, 95% CI 0.60–1.72, *p* = 0.95, *I*2 = 0%)
AS + IV colistin versus IV colistin alone	Significant; 3 studies, 415 patients (OR = 2.12, 95% CI 1.40–3.20, *p* = 0.0004, *I*2 = 0%)	Not significant; 2 studies, 242 patients (OR = 1.29, 95% CI 0.63–2.63, *p* = 0.48, *I*2 = 43%)	Not significant; 3 studies, 415 patients (OR = 0.75, 95% CI 0.50–1.11, *p* = 0.15, *I*2 = 0%)
Colistin-combined therapy versus colistin monotherapy	Not significant; 5 studies, 245 patients (OR = 1.38, 95% CI 0.81–2.33, *p* = 0.23, *I*2 = 0%)	Not significant; 4 studies, 212 patients (OR = 1.49, 95% CI 0.79–2.83, *p* = 0.22, *I*2 = 0%)	Not significant; 2 studies, 123 patients (OR = 0.48, 95% CI 0.22–1.03, *p* = 0.06, *I*2 = 0%)
C. Valachis et al. [78]			
Adjunctive AS colistin versus IV colistin	Significant; 8 studies, 690 patients (OR, 1.57; 95%CI, 1.14–2.15; *p* = 0.006)	Significant; 7 studies, 479 patients (OR, 1.61; 95%CI, 1.11–2.35; *p* = 0.01)	Not significant; 7 studies, 668 patients (OR, 0.74; 95%CI, 0.54–1.01; *p* = 0.06)
AS colistin monotherapy versus IV colistin	Not significant; 2 studies, 31 patients	Not significant; 2 studies, 31 patients	-

*AS* aerosolized, *IV* intravenous.

### Future roadmap for optimization of the clinical use of colistin—‘1st International Conference on Polymyxins,’ Prato, Italy, 2013

With the aim of better understanding of the current use of polymyxins, including factors affecting the safe and effective use of them, and identify research areas to fill gaps in existing knowledge, delegates from 27 countries from various specialties attended ‘The 1st International Conference on Polymyxins’ held in Prato, Italy, on 2–4 May, 2013. The consensus panel members identify high-priority issues, some of them are as follows [79]:

- ▪ Uniformity in expression of the amount of drug in a parenteral vial by different manufacturers should be expressed as mg of colistin base activity (CBA) or number of International Unit (IU)
- ▪ Uncertainties regarding susceptibility testing and breakpoints (currently under review jointly by the Clinical and Laboratory Standards Institute, CLSI and the European Committee on Antimicrobial Susceptibility Testing, EUCAST)
- ▪ Need of therapeutic drug monitoring in routine clinical practice (to maximize bacterial killing and minimize side effects in individual patients)
- ▪ Suggested research areas are prospective studies using therapeutic drug monitoring, pharmacokinetic studies in special patient populations, combination versus monotherapy, aerosol drug delivery alone, or in combination with intravenous route for the treatment of pneumonia

## Conclusions

In recent time, the uses of colistin to treat lung infection due to MDR gram-negative bacteria have been increased throughout the world, with little understanding of PK/PD of colistin. The usual dose of 3 MIU of colistin every 8 h intravenously achieves plasma colistin concentration just above the MIC level, and it took at least 48 h to reach the steady plasma colistin level. These finding stresses to use high loading (9 or 12 MIU of CMS) as well as high maintenance dose (4.5 MIU every 12 h or 9 MIU of CMS every 24 h) of intravenous colistin, but there is scarcity of clinical studies with these doses. Using colistin with carbapenems has been found to have a synergistic action, though there is no strong evidence for using colistin/carbapenem combination therapy. On the other side, having PAE and heteroresistance phenomenon, especially for *Acinetobacter* infection, small-interval doses regimens of colistin may have better efficacy and reduces the chance of development of resistant against colistin. Currently, available evidences throw highlight that different doses and/or dose intervals may be required for different types of gram-negative infections and warrant further studies for better understanding about PK/PD of colistin and its clinical applications. It is not only suboptimal plasma concentration of colistin but also poor lung tissue concentration, which has been demonstrated in recent studies, poses major concern in using intravenous colistin. Inhaled colistin as an adjunct treatment for lung infection due to MDR gram-negative bacteria have been found promising in recent studies as well as in meta-analysis.
